# How Can Prosocial Behavior Be Motivated? The Different Roles of Moral Judgment, Moral Elevation, and Moral Identity Among the Young Chinese

**DOI:** 10.3389/fpsyg.2018.00814

**Published:** 2018-05-28

**Authors:** Wan Ding, Yanhong Shao, Binghai Sun, Ruibo Xie, Weijian Li, Xiaozhen Wang

**Affiliations:** ^1^College of Teacher Education, Zhejiang Normal University, Jinhua, China; ^2^Faculty of Psychology, Beijing Normal University, Beijing, China

**Keywords:** prosocial behavior, moral judgment, moral elevation, moral identity, moderated mediation

## Abstract

Prior research has shown that moral judgment, moral elevation, and moral identity contribute to prosocial behavior. However, how these three motivating factors interact in predicting prosocial behaviors is not yet clear. The current study proposed and examined a moderated mediation model to illustrate the specific process of how prosocial behavior is motivated by these factors. A total of 264 Chinese undergraduate and graduate students participated in the present study (140 females; age range 17–26, *M* = 20.25, *SD* = 1.57). Moral judgment competence, intensity of moral elevation, and moral identity were measured by self-reported scales, and the tendency to engage in prosocial behavior was assessed in a simulated “Ask for help” situation. The multiple regressive results showed that moral elevation mediated the effect of moral judgment on prosocial behavior, and moral identity moderated this mediation through interacting with moral elevation. However, within the proposed model, the mediating effect of moral elevation was stronger in women than in men, while the moderating role of moral identity appeared only in women. These findings imply different methods for men and women to enhance their prosocial behaviors, including the need to pay more attention to developing moral reasoning in men while putting more emphasis on evoking moral emotion and moral traits in women. Together, these results supported the assumptive model and provided a comprehensive framework to explain prosocial behaviors.

## Introduction

Prosocial behavior, an important form of moral behavior (Fabes et al., 1999), is essential for maintaining positive social relationships and promoting social adjustment. Prosocial behavior was defined as behavior through which people benefit others (Eisenberg, 1982), including helping, cooperating, comforting, sharing, and donating (Eisenberg and Fabes, 1998; Greener and Crick, 1999). However, people in China are being recognized as reluctant to exhibit prosocial behaviors. The annual global survey published by the British Charities Aid Foundation (Charities Aid Foundation, 2015, 2016) showed China to have the lowest level of helping behaviors. Furthermore, the overall score on the World Giving Index for the Chinese was 144th place out of 145 countries in 2015, and 140th out of 140 countries in 2016 with only 23% (2015) and 24% (2016) of the population reporting to have helped a stranger in the past month (Charities Aid Foundation, 2015, 2016). Therefore, it is necessary to further understand the moral underpinnings of prosocial behaviors in China.

In the last few decades, researchers have focused on identifying factors that motivate prosocial behavior (Grant and Mayer, 2009). An extensive body of research has identified some important individual predictors of prosocial behavior (Hardy, 2006). These individual predictors fell into three categories: moral cognition (i.e., moral reasoning or moral judgment) (Kohlberg, 1969), moral emotion (e.g., moral elevation) (Haidt, 2003), and moral identity (Aquino and Reed, 2002; Blasi, 2004; Hardy, 2006). However, the existing research mainly emphasized on finding one or two moral motivations, then the limitations have emerged. For one thing, any single motivation is not sufficient to explain prosocial behavior. For example, effects of moral judgment have been shown to vary (Bickman, 1972; Walker, 2004; Carlo, 2005). For another, the specific role of each motivation and their interplays underlying moral conduct are not fully understood. These limitations suggest that all three moral motivations and their simultaneous interaction should be taken into consideration to explain prosocial conduct from a comprehensive perspective. Based on prior research, moral cognition, moral elevation, and moral identity are all positively related to prosocial behavior (Blasi, 2004; Carlo, 2005; Algoe and Haidt, 2009). Besides, the elevation may mediate the effect of moral judgment on prosocial behavior (Algoe and Haidt, 2009), and moral identity may play a moderating role in motivating moral conduct (Aquino et al., 2011).

### Moral Judgment and Prosocial Behavior

Moral judgment competence is the ability to make moral judgments (i.e., based on inner moral principles) and to act according to these judgments (Kohlberg, 1964). It reflects a person’s degree of moral cognitive development. According to Kohlberg (1976), moral cognitive development occurs at three levels, with each level having two distinct stages. These stages reflect the level of moral reasoning, which allows one to distinguish right from wrong actions and drives moral actions. Because of its role in moral conduct, moral judgment has long been the focus of research on morality.

Kohlberg’s (1969) cognitive developmental theory argued that moral judgment can inherently motivate moral action (Kohlberg, 1969; Smetana, 2006). As moral judgment capacities mature, one’s moral principles develop as well. It was further proposed that individuals grow to apply their moral principles to make judgments and guide actions. Thus, behavior tends to be consistent with their moral judgments as they mature. Numerous empirical studies examined moral judgment’s role in predicting prosocial behavior, and the findings varied. Some studies revealed a positive association between moral judgment and helping behavior (Eisenberg, 1986; Carlo, 2005). However, other studies also showed that moral judgment only explained 10% of the variance in moral conduct (Blasi, 1980; Walker, 2004). According to Bickman’s (1972) research, the relationship between moral judgment and prosocial behavior is weak. Specifically, 97% of participants declared they had the environment in mind, whereas just afterward, only 2% picked up garbage that had been left on the ground near a trashcan (Bickman, 1972). This disparity between attitudes and actions indicates the limitation of cognitive motivation.

### Moral Elevation and Prosocial Behavior

Moral elevation is one of the positive moral emotions triggered by seeing someone perform a virtuous act (Haidt, 2003). Moral elevation shares characteristics with other moral emotions and consists of a suite of responses that motivate prosocial actions, and tendencies (Haidt, 2003). The related components include thoughts, feelings, motivations, and physical changes (Izard, 1991; Ekman, 1992). The motivations associated with moral elevation are the desire to become a better person and the wish to open one’s heart to others (Keltner and Haidt, 2003; Silvers and Haidt, 2008). Algoe and Haidt (2009) also showed that moral elevation involves emotional experiences such as a feeling of being inspired or uplifted and physical sensations such as a feeling of warmth in the chest or a lump in the throat. Furthermore, thoughts related to moral elevation are a positive view of humanity (Freeman et al., 2009; Aquino et al., 2011).

Like any other moral emotion, moral elevation may conduct as the primary source of moral motivation, motivating goodness or directly holding a person back from doing something bad directly (Hoffman, 2000; Jones and Fitness, 2008; Lai et al., 2014). Significant effects of moral elevation on helping behavior have been documented in a variety of studies. For instance, individuals who have experienced moral elevation are more likely to offer help and develop more life goals related to morality (Algoe and Haidt, 2009; Van de Vyver and Abrams, 2015). Freeman et al. (2009) showed that exposing people to acts of moral goodness led them to donate more money to charity. Thus, moral elevation has been regarded as an important force driving prosocial actions of individuals (Haidt, 2003; Schnall et al., 2010).

### Moral Identity and Prosocial Behavior

Moral identity is organized around a cluster of moral traits and reflects the degree to which being moral is important to the self (Aquino and Reed, 2002). The trait-based definition stems from Blasi’s (1984) contention that some moral traits (e.g., being caring or helpful) may be more central to one’s self-concept than others (e.g., being honest or generous). Because of an individual’s desire for consistency between one’s self-definition and actions, an individual who considers him/herself to be a moral person will pursue more moral ideals (e.g., offering help, caring for others’ feelings, never cheating) (Aquino et al., 2009). Thus, moral identity has also been regarded as a self-regulatory system to motivate moral action (Blasi, 2004; Aquino et al., 2009; Brooks et al., 2013).

Many empirical studies found moral identity to be significantly and positively correlated with prosocial actions (Aquino and Reed, 2002; Aquino et al., 2009). According to Aquino et al. (2009) studies, higher level of moral identity decreased the willingness of participants to lie for a job candidacy during a salary negotiation and increased the intentions to contribute to the public good. Researchers also found that people with a high moral identity reported greater tendency to engage in helping behavior (Hardy, 2006) and donate more money (Reed et al., 2007). Therefore, researchers argued that moral identity can be used to explain moral action (Hardy, 2006).

However, limitations have emerged when researchers have studied only one moral motivation. For example, effects of moral judgment have been shown to vary, and any single motivation is not sufficient to explain prosocial behavior. Hence, researchers are increasingly interested in the combined effects of moral predictors. Their research revealed that moral emotions mediated the association between moral cognition and moral conduct (Aquino et al., 2011; Schnall and Roper, 2012) and moral identity facilitated the process from moral cognition and moral emotion to prosocial behavior (Reynolds and Ceranic, 2007; Aquino et al., 2009, 2011).

### Interplay of Two Predictors in Motivating Prosocial Behavior

#### Mediating Role of Moral Elevation

Empirical studies have shown that moral judgment may guide person’s moral emotion, and subsequently, moral emotion provides the driving force leading to prosocial behavior. For example, the association between moral judgment and moral emotion was presented in motivating prosocial behavior (Haidt, 2001; Valdesolo and DeSteno, 2006). Huebner et al.’s (2009) meta-analysis suggested that moral emotion, frequently followed by moral judgment, might perform as a direct motivator of moral behavior. Prior studies have suggested that moral elevation can play its role as moral emotion in putting moral values into action. (Schnall et al., 2010). Specifically, moral elevation was found to put one’s moral standards into prosocial behavior (Algoe and Haidt, 2009; Aquino et al., 2011).

#### Moderating Role of Moral Identity

Some evidence showed that moral identity moderated the process from moral cognition or moral emotion to prosocial behavior (Reynolds and Ceranic, 2007; Aquino et al., 2009, 2011). Several studies showed that high moral identity facilitated the effect of moral judgment on prosocial conduct (Aquino and Reed, 2002; Reynolds and Ceranic, 2007). Studies also implicated the interaction between moral emotion and moral identity in predicting moral conduct (Aquino et al., 2009). Specifically, compared with people with low moral identity, the association between moral elevation and prosocial behavior was stronger for people with high moral identity. Thus, moral identity may promote the effect of moral judgment or moral elevation on prosocial behavior.

### Gender Differences in Prosocial Behaviors

Numerous research demonstrated the gender differences in prosocial behaviors (Gilligan, 1982; Spain, 2001; Eckel and Grossman, 2008). Most surveys found that more women than men engaged in volunteering and helping others (Eckel and Grossman, 2008; Bureau of Labor Statistics, 2009; Themudo, 2009). Bureau of Labor Statistics (2009) found that 30.3% of women volunteered during the previous year, compared with 23.3% of men. In experimental research, it was found that women are usually more likely to help others, although this behavior varies with the conditions of the experiment (Eckel and Grossman, 2008). Although there has been much research about the gender difference on prosocial behavior, the mechanism underlying helping behavior for each gender is not clear enough. Is there any difference between men and women or do they share the same mechanism? This remains to be an important issue.

Some studies have found the gender difference in moral motivations that predict helping behaviors (Lee et al., 1999; Einolf, 2011). Compared with men, women scored higher on moral traits (e.g., caring and prosocial role identity) and moral emotions (e.g., empathy and guilt) (Eagly and Crowley, 1986; Davis, 1994; Lee et al., 1999; Einolf, 2011). Kohlberg (1981) thinks that men have higher levels of the sophistication in moral reasoning, although this was not found in subsequent empirical research (Skoe et al., 2002). Given that men are always associated with impersonal reasoning and rule making while women are associated with caring, relationships, and helping others (Gilligan, 1982; Einolf, 2011), it is reasonable to conclude that moral judgment motivates prosocial behavior more often in men than in women, and moral elevation and moral identity result in prosocial behavior more frequently in women than in men.

### The Present Study

**FIGURE 1:**
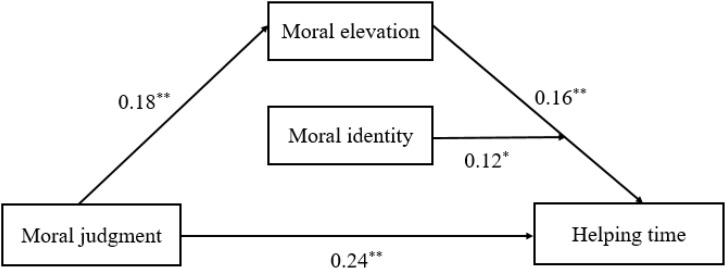
The moderated mediation models for total sample. ^∗^p < 0.05, ^∗∗^p < 0.01.

This study aimed to examine the specific roles of moral judgment, moral elevation, and moral identity within the comprehensive framework of morality. Based on the existing literature (Reynolds and Ceranic, 2007; Aquino et al., 2009, 2011; Schnall and Roper, 2012), we proposed a moderated mediation model which contained two key hypotheses (See **Figure 1**): (1) Moral elevation has a mediating effect on the relationship between moral judgment and prosocial behavior; (2) Moral identity has a moderating effect on prosocial behavior, interacting with moral judgment, moral elevation, or both moral judgment and elevation. Then, considering the gender differences in prosocial behaviors and moral motivations, we also examined whether men and women share the same mechanism. We predicted that the mediating effect of moral elevation and moderating effect of moral identity in women would be stronger than in men (Gilligan, 1982; Einolf, 2011).

## Materials and Methods

### Participants

The sample included 282 undergraduate and graduate students (156 female; age range 17–27, *M* = 20.28, *SD* = 1.59) from Zhejiang Normal University. Participants were recruited through public selective classes (available to students from all majors) and were paid ¥20 (about $3) for their involvement. Fifteen participants were excluded because of invalid questionnaires (involving the same, completely random, or contradictory item responses). Three additional participants were excluded because of suspicion about the study purpose. The final sample included 264 participants (140 female; age range 17–26, *M* = 20.25, *SD* = 1.57).

### Measures

#### Moral Judgment

The participants’ moral judgment competence was measured using the Chinese version of Moral Judgment Test with good validity on construction and content (C-MJT, Lind, 1978; Yang and Wu, 2006). The C-MJT consists of two sub-tests, and each test contains a dilemma story (Workers’ Dilemma and Doctor’s Dilemma) as well as 12 questions pertaining to agreement/disagreement with a suggested solution to the dilemma. The respondents were asked to evaluate the acceptability of the pro and con arguments presented in the C-MJT on an 8-point scale (- 4 = completely unacceptable, 4 = completely acceptable). The C-MJT is a unique measuring system without classical psychometric properties. Analysis according to five empirical criteria (*the order of preferences, quasi-simplex structure, cognitive-affective parallelism, equivalence of* pro and con arguments, *a difficult moral task*) derived from cognitive-developmental theory indicates good theoretical and pragmatic validity of the measure (Lind, 1978; Yang and Wu, 2006). The C-index reflects the extent to which a person’s judgments are acceptable with respect to the pro and con descriptions, representing an individual’s level of moral judgment. It is computed from the raw score of subjects according to Lind’s (1995) detailed process and ranges from zero (indicating an absence of any moral judgment competence) to 100 (indicating perfect judgment competence). Since C-index is used to examine the internal consistency (C-index) of a subject’s moral reasoning, it is obviously useless to check the validity of the test by relying on the classical test theory (Rest et al., 1997).

#### Moral Identity

The Chinese version of Moral Identity Scale (C-MIS) consists of internalization (reflecting the importance of possessing moral traits) and symbolization (reflecting the importance of demonstrating to others that they possessed moral traits) subscales (Aquino and Reed, 2002; Wan, 2009). In the current study, focusing on the chronic aspect of moral identity, we intended to test related theoretical predictions using the internalization subscale (Aquino et al., 2009). This questionnaire asked participants to imagine a person who possesses nine moral traits (e.g., helpful and caring) and then presented five items (e.g., “It would make me feel good to be a person who has these characteristics”). Each of the items was to be answered on a 7-point scale ranging from 1 (strongly disagree) to 7 (strongly agree). A higher average score on the five items indicated higher moral self-importance for each participant. In the current study, the Cronbach’s α for internalization subscale was 0.81.

#### Moral Elevation

We used the Chinese Version of the Moral Elevation Scale (C-MES, Haidt, 2003; Ding et al., 2014) to assess participants’ intensity of moral elevation. The C-MES consists of a description task pertaining to goodness (elevation elicited through the recall of morally virtuous acts) and 21 items to assess one’s degree of moral elevation. The C-MES includes four dimensions: eight items about elevating emotions and physiological responses (e.g., “moved,” “inspired,” “uplifted,” “warm feeling in chest”), four items about the desire to be a better person (e.g., “I want to be more like the person/people in the story”), five items about the tendency to open one’s heart to others (e.g., “I want to help others”), and four items about views on humanity (e.g., “The world is full of kindness and generosity”). Each of the items was to be answered on a 5-point scale ranging from 1 (strongly disagree) to 5 (strongly agree). Higher scores reflected a stronger sense of moral elevation among participants. In this study, the Cronbach’s α was 0.92 for the total scale, and were 0.85, 0.82, 0.80, and 0.70 for elevating emotions and physiological responses, desire to be a better person, tendency to open one’s heart to others, and views on humanity subscale, respectively.

#### The Tendency of Volunteering Behavior

A method revised from Schnall et al.’s (2010) measure was used to assess the tendency to engage in prosocial behavior. Specific operations were as follows. First, after completing the C-MES, participants were informed that the study had ended and that they would be paid ¥20 (about $3) for their participation. Then, they were asked to complete a payment receipt on the last page of the questionnaire, which contained a brief survey to assess their willingness to participate in an additional unpaid study (choose “yes” or “no”). It stated, “There is another survey for which we need your help, without any pay. Any amount of help would be greatly appreciated. You are free to decide whether you will be willing to help us and to choose the time you wish to spend on the survey before the survey starts.” The participants were asked to choose the time they would be willing to devote ranging from 0 to 120 min, at intervals of 10 min. According to Korsgaard et al.’s (2010) study, the experimenter should state that the participants (a) would receive no incentive for participating and (b) were not obligated to participate. Also, all participants who were ready to offer help were requested to provide their contact information for the additional study. This method was proved to be a valid assessment tool for evaluating participants’ voluntary helping behavior and had been used in several studies (Oswald, 2002; Korsgaard et al., 2010). Owing to this “Ask for help” situation being well developed in the prior study, the actual tendency of volunteering behavior could be measured.

### Procedure

The ethics committee of Zhejiang Normal University in China approved the protocol of this study. We also obtained a written consent from all participants, who in turn were given envelopes containing information about the measures. All materials and measures were completed anonymously in class. At the beginning of the survey, items on moral judgment and moral identity were presented, followed by 30 filler questions unrelated to morality (How many hours of sleep do you get every day? What did you have for lunch yesterday? Do you think that reading is more fun than watching television? Do you enjoy watching sport than playing?). Then, the participants completed a description task pertaining to goodness (elevation elicited through recall of morally virtuous acts). Next, the C-MES was used to measure the moral elevation of the participants. Last, all participants took part in a test of their prosocial intentions through a simulated “request for help” situation.

### Data Analysis

Before testing our predictions, we conducted descriptive and correlation analyses using SPSS 20.0 to describe the characteristics of the Chinese sample. Then, with controlling for gender (0 = male, 1 = female) (Gilligan, 1982), the proposed moderated mediation model for the total sample was examined in SPSS 20.0 with centered variables (Aiken et al., 1991), utilizing a multiple regression analysis process (Muller et al.’s, 2005). The regression analysis was conducted using the enter method. Bootstrap confidence intervals (CI) were computed for the regressions coefficients, and a 95% CI not containing 0 indicated a significant result (Erceg-Hurn and Mirosevich, 2008). Finally, to clarify whether the proposed model was identical for both male and female, the regression analyses for males and females were conducted separately.

## Results

### Descriptive Statistics and Correlation Analyses

The means, standard deviations, and Pearson’s correlation coefficients of the variables for male and female are presented in **Table 1**. The results showed that the prosocial behaviors were positively correlated with moral judgment (*r*_male_ = 0.27, *p* < 0.01; *r*_female_ = 0.29, *p* < 0.01) and moral elevation (*r*_male_ = 0.22, *p* < 0.01; *r*_female_ = 0.35, *p* < 0.01), regardless of the gender. However, prosocial behaviors were correlated with moral identity in women (*r*_female_ = 0.18, *p* < 0.01), but not in men (*r*_male_ = 0.10, *p* > 0.05).

**Table 1:** Descriptive statistics and correlation matrix.

	Male *(n* = 124)					Female (*n* = 140)				
	*M*	*SD*	1	2	3	*M*	*SD*	1	2	3
(1) Moral judgment	25.49	18.02	–			28.20	15.57	–		
(2) Moral identity	26.29	5.24	0.09	–		29.42	4.23	0.13	–	
(3) Moral elevation	80.25	10.91	0.16**	0.11	–	83.51	12.00	0.20**	0.10	–
(4) Helping time	43.72	19.72	0.27**	0.10	0.22**	45.35	18.37	0.29**	0.18**	0.35**

*^∗^p < 0.05. ^∗∗^p < 0.01.*

### Linear Regression Analysis

#### Mediation Effect

The results in **Table 2** and **Figure 1** show that moral judgment not only predicted prosocial behavior directly (*B* = 0.28, *SE* = 0.05, 95% *CI* = 0.18–0.37), but also had a significant indirect effect on prosocial behavior through moral elevation. To be specific, the paths from moral judgment to moral elevation and from moral elevation to prosocial behavior were significant (*B* = 0.18, *SE* = 0.05, 95% *CI* = 0.09–0.29; *B* = 0.16, *SE* = 0.05, 95% *CI* = 0.05–0.26). The direct effect of moral judgment on prosocial behavior was reduced from 0.275 to 0.244 when moral elevation was added to the model. This indicated a partial mediating effect of moral elevation in the predicting of moral judgment on prosocial behavior, and this mediating effect comprised 10.9% (0.03/0.275) of the total effect. Moreover, the adjusted R^2^ of predicting helping time increased from 0.08 to 0.18 when moral elevation was added. That is, moral elevation led to an additional R^2^ value with 10%.

**Table 2:** Least squares regression results for the total sample.

Predictor variables	The first step			The second step			The third step		
	Helping time			Moral elevation			Helping time		
	*B*	*SE*	95%*CI*	*B*	*SE*	95%*CI*	*B*	*SE*	95%*CI*
X: Moral judgment (MJ)	0.28^∗∗^	0.05	[0.18, 0.37]	0.18^∗∗^	0.05	[0.09, 0.29]	0.24^∗∗^	0.05	[0.14, 0.34]
Mo: Moral identity (MI)	0.05	0.05	[-0.05, 0.15]	0.09	0.05	[-0.01, 0.20]	0.07	0.05	[-0.03, 0.18]
XMo: MJMI	0.02	0.05	[-0.07, 0.12]	0.01	0.05	[-0.09, 0.11]	0.01	0.05	[-0.10, 0.11]
Me: Moral elevation (ME)	–		–	–		–	0.16^∗∗^	0.05	[0.05, 0.26]
MeMo: MEMI	–		–	–		–	0.12^∗^	0.06	[0.01, 0.25]
Adj *R*^2^		0.08			0.05			0.14	
*F*		10.04			4.44			8.68	

*Mo, moderator variable; Me, mediator variable. ^∗^p < 0.05, ^∗∗^p < 0.01.*

#### Moderated Mediation

**FIGURE 2:**
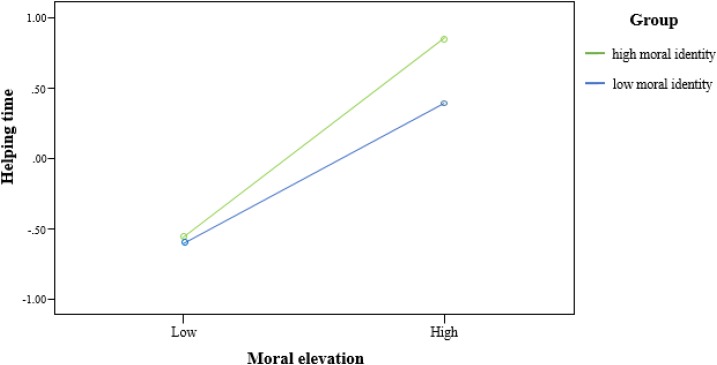
The relationship between moral elevation and helping time for high and low levels of moral identity.

As **Table 2** and **Figure 1** show, moral identity had a facilitating effect on the mediation by interaction with moral elevation (*B* = 0.12, *SE* = 0.06, 95% *CI* = 0.01–0.24). To present the moderating role of moral identity in motivating moral conduct, we plotted an interaction in **Figure 2** at different levels of moral elevation and moral identity (1 SD above and below the mean for high and low levels). Compared with low-MI people, high-MI people would devote more time to helping others when they were evoked with the same strong level of moral elevation (*M*_high-MI_ = 0.85, *M*_low-MI_ = 0.39, *t*_(35)_ = 2.68, *p* < 0.01, Cohen’s *d* = 0.46).

#### Gender Differences

**FIGURE 3:**
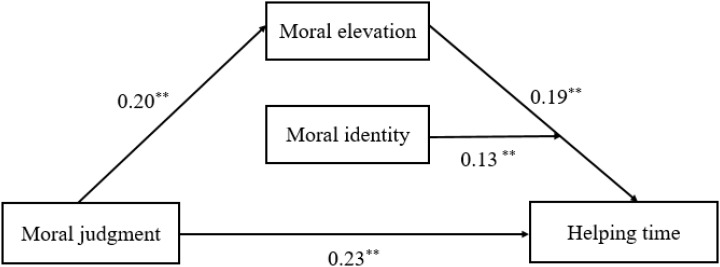
The moderated mediation models for women. ^∗^p < 0.05, ^∗∗^p < 0.01.

**FIGURE 4:**
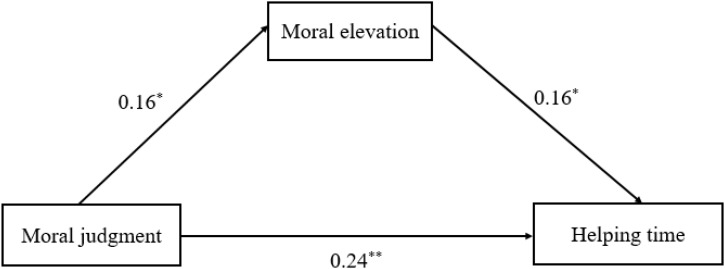
The moderated mediation models for men. ^∗^p < 0.05, ^∗∗^p < 0.01.

The regression analysis showed a similar mechanism in women and men, with a slight difference in effect size of moral elevation and role of moral identity. For women (see **Table 3** and **Figure 3**), moral judgment had a significant indirect effect on prosocial behaviors through moral elevation (*B* = 0.20, *SE* = 0.05, 95% *CI* = 0.10–0.30; *B* = 0.19, *SE* = 0.05, 95% *CI* = 0.09–0.28), and moral identity facilitated this mediation (*B* = 0.13, *SE* = 0.06, 95% *CI* = 0.02–0.24). For men (see **Table 4** and **Figure 4**), moral elevation also mediated the effect of moral judgment on prosocial behaviors (*B* = 0.16, *SE* = 0.05, 95% *CI* = 0.04–0.28; *B* = 0.16, *SE* = 0.05, 95% *CI* = 0.05–0.27), while the moderating effect of moral identity did not appear (*B* = 0.09, *SE* = 0.06, 95% *CI* = -0.01–0.19). Additionally, the mediating effect of moral elevation occupied 9.3% (0.0256/0.276) of the total effect in men and 13.3% (0.038/0.285) in women, indicating a stronger mediating role of moral elevation in women than in men.

**Table 3:** Least squares regression results for women.

Predictor variables	The first step			The second step			The third step		
	Helping time			Moral elevation			Helping time		
	*B*	*SE*	*95%CI*	*B*	*SE*	*95%CI*	*B*	*SE*	*95%CI*
X: Moral judgment (MJ)	0.29^∗∗^	0.05	[0.19, 0.39]	0.20^∗∗^	0.05	[0.10, 0.30]	0.23^∗∗^	0.05	[0.13, 0.33]
Mo: Moral identity (MI)	0.06	0.05	[-0.04, 0.16]	0.07	0.05	[-0.01, 0.15]	0.08	0.05	[-0.02, 0.18]
XMo: MJMI	0.02	0.05	[-0.07, 0.12]	0.02	0.05	[-0.08, 0.12]	0.01	0.05	[-0.10, 0.11]
Me: Moral elevation (ME)	–		–	–		–	0.19^∗∗^	0.05	[0.09, 0.28]
MeMo: MEMI	–		–	–		–	0.13^∗^	0.06	[0.02, 0.24]
Adj *R*^2^		0.09			0.06			0.15	
*F*		10.35			4.78			9.24	

*Mo, moderator variable; Me, mediator variable. ^∗^p < 0.05, ^∗∗^p < 0.01.*

**Table 4:** Least squares regression results for men.

Predictor variables	The first step			The second step			The third step		
	Helping time			Moral elevation			Helping time		
	*B*	*SE*	*95% CI*	*B*	*SE*	*95%CI*	*B*	*SE*	*95% CI*
X: Moral judgment (MJ)	0.28**	0.05	[0.19, 0.38]	0.16*	0.05	[0.04, 0.28]	0.24**	0.05	[0.13, 0.35]
Mo: Moral identity (MI)	0.04	0.05	[-0.05, 0.13]	0.09	0.05	[-0.01, 0.19]	0.06	0.05	[-0.03, 0.15]
XMo: MJMI	0.04	0.05	[-0.06, 0.14]	0.01	0.05	[-0.09, 0.11]	0.03	0.05	[-0.07, 0.13]
Me: Moral elevation (ME)	-		–	-		–	0.16*	0.05	[0.05, 0.27]
MeMo: MEMI	-		–	-		–	0.09	0.06	[-0.01, 0.19]
Adj *R*^2^		0.08			0.05			0.14	
*F*		10.57			4.11			8.09	

*Mo, moderator variable; Me, mediator variable. ^∗^p < 0.05, ^∗∗^p < 0.01.*

## Discussion

The current study examined a comprehensive model of prosocial conduct among a Chinese young adult sample by simultaneously integrating the roles of moral judgment, moral elevation, and moral identity. Consistent with hypotheses, the results showed the mechanism underlying prosocial conduct and illuminated the different roles of the three sources of moral motivation in predicting prosocial behaviors. Specifically, it was found that moral judgment motivates prosocial behavior directly as well as through moral elevation. Moral identity facilitates the process from moral judgment to prosocial behavior by interacting with moral elevation in the indirect path. Furthermore, although both women and men share similar mechanism, we found that the mediating effect of moral elevation is stronger in women than in men while moderating role of moral identity only shows in women. Our findings clearly illustrated the direct and indirect pathways of how prosocial behavior is driven by moral cognition, emotion, and identity in men and women. Thus, our findings support a comprehensive view of morality and highlight the interplay between the three sources of moral motivation.

First, moral elevation mediated the predicting of moral judgment on prosocial behavior. These results supported the assumption that moral elevation plays a role in putting moral values into action. This helped to explain the varied findings for effect of moral thoughts on moral actions (Bickman, 1972; Walker, 2004; Carlo, 2005). That is, people may not always engage in prosocial behaviors when they have moral thoughts; however, people will engage in prosocial behaviors when their moral emotions help bridge the gap between moral values and actual behaviors (Hoffman, 2000; Ayal et al., 2015). Thus, evoking moral elevation not only enables the access to prosocial behavior when moral thoughts are present, but also contributes to accumulating the effects of moral judgment and moral elevation to motivate prosocial behavior. Overall, the mediation model describes pathways from moral judgment to moral action, highlights the vital importance of moral elevation in the process, and implicates methods of increasing moral actions.

Second, we also found moral identity moderated the mediation by interacting with moral elevation, which helps to clarify the specific role of moral identity in moral self-regulation (Blasi, 2004; Aquino et al., 2009). Specifically, moral identity, a relatively stable moral trait, had no direct effect on prosocial actions when the three motivations were all considered in the full model. Within the comprehensive framework, we only found that moral identity motivated prosocial actions through facilitating moral elevation’s effect. That is, compared with Low-MI people, those with High-MI were more likely to offer help in moral situations after they saw uncommon goodness. One possible explanation for this moderation is the assimilation effect of upward social comparisons. Based on the gap between the self and the comparison target, people may feel a sense of self-inspiration or self-stagnation (Algoe and Haidt, 2009; Yip and Kelly, 2013). To be specific, High-MI people made the social comparison toward someone they felt to be similar in pursuit of goodness, inducing their self-enhancement through assimilation of others’ virtues. In contrast, although low-MI individuals feel moved, warm, and have a positive view of humanity when they witness virtue, the large gap between moral pursuits of self and the model weakened the motivation to emulate the model and do good deeds. In short, the similarity on moral identity urges one to follow the model while the large gap on moral identity makes one hesitate to emulate the prosocial action. This assumption may help to explain why there was a stronger effect of moral elevation on prosocial behavior for high-MI people compared to low-MI people. However, this topic requires further examination in future research.

Furthermore, although the proposed model was generally identical in both males and females, gender differences were subtly presented on the effect size of moral elevation and the role of moral identity. The mediating effect accounts for more of total effect in women (13.3%) than in men (9.3%). This means a relatively weaker proportion of moral judgment’s direct effect in total motivating effect among women than men, although there was no obvious difference on absolute size of the direct effect between them. This finding supported the assumption that women often offer help for being evoked with moral emotions, while men often help others because of their moral judgment (Skoe et al., 2002; Einolf, 2011). In contrast with women, the moderating role of moral identity was not shown within men. Considering that men are always associated with impersonal reasoning and behave in accordance with their judgment or value (Kohlberg, 1981; Einolf, 2011), we presumed moral identity motivates prosocial behaviors through other unknown ways (e.g., working by blending with moral judgment). Future research is needed to clarify this issue.

The limitations of the present study should be addressed here. Firstly, moral elevation was evoked by having participants recall a virtue in their minds; this method is more abstract than one that involves showing actual videos of goodness, which may induce moral emotion through audio and visual stimuli. Hence, future research should focus on developing new video material to evoke moral elevation effectively. Secondly, self-assessment of moral elevation is limited in its ability to show the duration and dynamic feelings of emotion. In view of this, the development and use of physiological or brain imaging techniques is encouraged in future research to record the dynamics of elevation feelings. Thirdly, not having considered participants’ other characteristics (e.g., age, grade, or academic type) except for gender, we didn’t get a strict-enough gender match for the subjects. Future research with a stricter gender match should be conducted to examine the compared the mechanism underlying prosocial behavior. Lastly, the generalizability of the findings may be challenged because all participants were recruited from China. The eastern and western cultural differences pertaining to this proposed model need to be examined in future studies.

In summary, our moderated mediation model examined the specific roles of moral judgment, moral elevation, and moral identity within the comprehensive framework of morality. Moral elevation mediated the predicting of moral judgment on prosocial behaviors, and moral identity moderated this mediation within the moral self-regulation system. Furthermore, although both women and men share similar mechanisms, women often offer help for being evoked with moral emotions, while men often help others because of their moral judgment. From an applied perspective, the present findings imply methods to increase prosocial behaviors. On the one hand, practical efforts should concentrate on arousing moral emotions in individuals when moral judgment is sufficiently mature. Moreover, works on promoting individuals’ moral identity should encouraged to facilitate the effect of moral emotions on moral actions. On the other hand, the approaches to enhance prosocial behaviors differ by genders. If possible, more attentions could be paid to developing men’s moral reasoning, and evoking women’s moral emotion and moral traits to enhance their prosocial behaviors, respectively.

## Author Contributions

BS, XW, WD, and WL conceived and designed the experiments. XW and WD performed the experiments. WD and YS analyzed the data. WD, YS, and RX contributed to the writing of the manuscript.

## Conflict of Interest Statement

The authors declare that the research was conducted in the absence of any commercial or financial relationships that could be construed as a potential conflict of interest.
